# Machine Learning Identifies Shared Regulatory Mechanisms of Genes Associated with Ferroptosis in Major Depressive Disorder and Inflammatory Bowel Disease

**DOI:** 10.3390/genes16091111

**Published:** 2025-09-19

**Authors:** Jiyuan Shi, Luojin Wu, Lingxi Li, Ye Liu, Yuxuan Lu, Mengmeng Sang, Liming Mao

**Affiliations:** 1 Department of Immunology, School of Medicine, Nantong University, 19 Qixiu Road, Nantong 226001, China; 2331310021@stmail.ntu.edu.cn (J.S.); 2431310023@stmail.ntu.edu.cn (L.W.); 2331110020@stmail.ntu.edu.cn (L.L.); 2331110062@stmail.ntu.edu.cn (Y.L.); 2431110598@stmail.ntu.edu.cn (Y.L.); 2 Basic Medical Research Center, School of Medicine, Nantong University, Nantong 226019, China

**Keywords:** ferroptosis, major depressive disorder, inflammatory bowel disease, machine learning, molecular docking, molecular dynamics analysis, single cell analysis

## Abstract

Background: Major depressive disorder (MDD) and inflammatory bowel disease (IBD) form a “bidirectional vicious cycle” through the gut–brain axis: psychological and emotional abnormalities can induce intestinal inflammation, while intestinal inflammation can in turn exacerbate mental health disorders. Ferroptosis is an iron-dependent form of regulated cell death that is driven by lipid peroxidation. Although this process has been molecularly defined in recent years, its role in the context of IBD and MDD remains insufficiently investigated. This study investigates the molecular roles of ferroptosis-related genes (FRGs) in both conditions and explores potential therapeutic strategies targeting these genes. Methods: We first identified differentially expressed FRGs (DE-FRGs) by comparing normal and disease samples. Subsequently, we screened for DE-FRGs in both IBD and MDD and named them Co-DEGs. Correlation analyses of these co-FRGs were performed, including comparisons between disease and control groups, as well as associations between Co-DEGs and immune cell infiltrations. Four distinct machine learning algorithms were employed to identify the core Co-DEGs associated with both IBD and MDD. Moreover, analyses of drug sensitivity, molecular docking, and molecular dynamics simulations were carried out to predict potential therapeutic agents for both conditions. Finally, single-cell sequencing analysis was also performed. Results: We identified 29 Co-DEGs in both IBD and MDD. Machine learning analysis identified *RPL8* as a key common biomarker exhibiting a consistent expression trend in both diseases. A predictive approach integrating molecular docking and molecular dynamics simulations indicated that LE135, a compound targeting *RPL8*, is the most promising therapeutic candidate. Conclusions: These discoveries enhance the understanding of the shared and distinct regulatory mechanisms of FRGs in gut–brain axis disorders. We have pinpointed key biomarkers and predicted potential therapeutic agents that may offer dual-targeting strategies for both IBD and MDD.

## 1. Introduction

The occurrence and development of inflammatory bowel disease (IBD), including ulcerative colitis (UC) and Crohn’s disease (CD), are affected by a mix of immune reactions, environmental elements, genetic susceptibilities, and changes in the intestinal microbiota [1]. The global prevalence of IBD is increasing, and many patients experience delays in diagnosis and access to effective therapies, highlighting an urgent need for the development of predictive biomarkers to assess therapeutic response, with the aim of reducing healthcare costs and alleviating disease burdens [2]. Depression is a highly prevalent and debilitating mental disease that frequently manifests during adolescence [3]. Patients with IBD often develop mental health problems, including anxiety and depression, which may result from gut–brain axis interactions, the long-lasting nature of the disease, diminished quality of life, and limitations in social functioning [4]. In IBD, disturbances of the gut microbiota can alter immune activity and brain function through microbiota–gut–brain signaling, promoting anxiety and depressive symptoms [5,6]. These symptoms may develop into major depressive disorder (MDD), which is marked by somatic, emotional, cognitive, and behavioral disturbances [7,8]. In the last thirty years, a large body of evidence has built up to back the idea that the improper regulation of inflammatory processes is crucial to the pathogenesis of depression [9].

Ferroptosis, a distinct form of regulated cell death triggered by iron-dependent lipid peroxidation, has recently been recognized as a critical regulatory mechanism in human physiology and pathology [10]. Experimentally, ferroptosis can be induced by directly inhibiting glutathione peroxidase 4 (GPX4), a key antioxidant enzyme in mammals, which plays a prominent role in preventing ferroptotic cell death by protecting cells against harmful lipid peroxidation [11,12]. Ferroptosis can be induced by compounds such as erastin and sulfasalazine (SSZ), which act through the inhibition of system XC−. Additionally, some compounds such as RSL3 and statins can induce ferroptosis by suppressing the activity of GPX4. In contrast, inhibitors of ferroptosis, such as ferrostatin-1, liproxstatin-1, and α-tocopherol, exert their protective effects by blocking the lipid peroxidation cascade. Moreover, agents such as deferoxamine and deferiprone inhibit ferroptosis by targeting other cellular pathways [13]. Accumulating evidence indicates that ferroptosis plays a significant role in the pathogenesis of various human diseases [14,15]. Programmed cell death (PCD) pathways like apoptosis and ferroptosis are significantly implicated in the progression of IBD, mainly through regulating the viability of intestinal immune cells and epithelial cells [16]. Ferroptosis has been observed in a range of human diseases, including IBD and MDD. Notably, disturbances in iron and lipid metabolism, consistent with the hallmark features of ferroptosis, including iron deposition and lipid peroxidation (LPO), are increasingly recognized as key regulatory mechanisms in the development of both IBD and MDD [17]. However, up to the present, there has been a lack of research that has pinpointed ferroptosis-related genes (FRGs) with the ability to exert simultaneous impacts on both IBD and MDD. Moreover, no therapeutic agents have been developed that can effectively regulate these FRGs to modulate the advancement of these two distinct yet potentially interconnected diseases.

To clarify the role of FRGs in MDD and IBD and to discover potential therapeutic agents, we applied bioinformatics to investigate shared ferroptosis-related mechanisms. Our analysis highlighted *RPL8* as a potential biomarker and identified BRD1812 as a promising candidate drug for both disorders. These results offer novel insights into the pathogenic mechanisms of IBD and MDD and may contribute to the development of novel treatment strategies.

## 2. Materials and Methods

### 2.1. Data Source

All datasets utilized in this study were sourced from the GEO database. For both UC and CD datasets, the samples were collected from colon biopsies. Specifically, the datasets utilized for UC analysis are as follows: GSE13367, consisting of 20 control samples and 34 UC samples [18]; GSE24287, which includes 25 control samples and 27 UC samples [19]; and GSE179285, containing 31 control samples and 55 UC samples [20]. As for CD, the applied datasets are as follows: GSE20881, which contains 73 control samples and 99 CD samples [21]; GSE24287, including 25 control samples and 47 CD samples [19]; and GSE179285, with 31 control samples and 168 CD samples [20]. For major depressive disorder (MDD), two datasets were used for blood samples: GSE98793, which is based on peripheral blood samples (64 control samples and 128 MDD samples) [22]; and GSE19738, derived from whole blood samples (66 control samples and 66 MDD samples) [23]. In addition, MDD-related datasets from human brain tissues were also used in this study: GSE54568, obtained from the prefrontal cortex, with 15 control samples and 15 MDD samples [24]; GSE54571, from the anterior cingulate cortex, including 13 control samples and 13 MDD samples [24]; and GSE54564, sourced from the amygdala tissue, containing 21 control samples and 21 MDD samples [24]. In total, 264 FRGs (Supplementary Table S1) were obtained from the FerrDB repository (http://www.zhounan.org/ferrdb/; accessed on 29 May 2025). The three CD datasets, three UC datasets, and two MDD whole blood datasets were merged separately, and batch effects were corrected using the ComBat algorithm in the “sva” R package (v3.52.0) [25]. PCA plots confirmed that batch effects were effectively corrected across different datasets (Supplementary Figure S1).

### 2.2. Identification of DE-FRGs

DE-FRGs were identified between normal and disease samples in CD, UC, and different MDD tissues using the Wilcoxon test (*p* < 0.05). Differential expression results were then visualized with a heatmap using the pheatmap package (v1.0.13).

### 2.3. Correlation Analysis Between Co-DEGs, and Between Co-DEGs and Immune Cells

Pearson’s correlation coefficients were computed to explore potential associations among Co-DEGs. The results were visualized using the “corrplot” package (v0.95). Additionally, Pearson’s correlation coefficients were calculated to investigate potential associations between Co-DEGs and immune cells, with visualizations generated using the “ggplot2” package (v3.5.1).

### 2.4. Machine Learning Screening of Potential Biomarker

In this study, four machine learning algorithms, including Support Vector Machine (SVM) [26], Random Forest (RF) [27], Generalized Linear Model (GLM) [28], and eXtreme Gradient Boosting (XGB) [29], were utilized to predict the significance of Co-DEGs in distinguishing normal and disease samples. The top 10 genes for each machine learning algorithm were selected as important genes. Genes that were deemed important by two or more machine learning algorithms were regarded as key factors contributing to the occurrence of diseases.

### 2.5. Differential Analysis and Gene Set Variation Analysis (GSVA) Between RPL8+ and RPL8− Groups

Samples were stratified into *RPL8+* and *RPL8*− groups based on *RPL8* expression levels. The results of differential expression analysis were shown as a volcano plot using the ggplot2 package (v3.5.1), and differentially expressed genes were named *RPL8*-DEGs. Subsequently, Gene Set Variation Analysis (GSVA)—an algorithm for evaluating gene set (pathway) activity based on gene expression data—and the differential analysis algorithm from the limma package were applied to compare pathway activities between the *RPL8+* and *RPL8*− groups. Finally, the differential analysis results were visualized in the form of a bar plot using the ggplot2 package (v3.5.1).

### 2.6. GO Enrichment and KEGG Pathway Analysis

Functional enrichment analysis of *RPL8*-DEGs was performed to assess the potential biological functions of the genes and the related pathways using the cluster Profiler R package (v4.10.1) [30].

### 2.7. Drug Prediction

The oncoPredict package (v0.2) was applied to estimate drug IC50 values using data from the Cancer Therapeutics Response Portal (CTRP). These predictions were generated based on the expression profiles of Co-DEGs between normal samples and diseased samples in MDD and IBD. Furthermore, the associations between predicted IC50 values of candidate drugs and the expression levels of Co-DEGs were assessed.

### 2.8. Molecular Docking Analysis

The protein structure was obtained from the Protein Data Bank (https://www.uniprot.org/; accessed on 1 May 2025) and used as the docking model after removing ligands. Drug structures were collected from ZINC15 (https://zinc15.docking.org; accessed on 1 May 2025) and the PubChem Small Molecule Database (https://pubchem.ncbi.nlm.nih.gov/; accessed on 1 May 2025). Molecular docking and receptor–drug visualization were performed using PyMol software (v3.0.5), and binding free energies were calculated accordingly.

### 2.9. Molecular Dynamics (MD) Simulation

MD simulation is an approach used to evaluate the stability and dynamic interactions of proteins and/or between protein and their ligands [31,32,33,34]. Here, the GROMACS (v2023) MD simulation package was employed to conduct the MD simulation of proteins and their ligands [35]. Protein topologies and the parameters were generated with the AMBER99SB force field, whereas ligand topologies and the parameters were obtained via the Chimera server. In this study, structural properties evaluated were RMSD, radius of gyration (Rg), solvent-accessible surface area (SASA), RMSF, hydrogen bonding, secondary structure, and angular and distance measurements. Simulation trajectories were performed using CMD (v10.0.26100.2894), and plots were generated with DuIvyTools (v0.5.0) (https://duivytools.readthedocs.io/; accessed 20 May 2025).

### 2.10. Single-Cell RNA Sequencing Analysis

Here, we selected the single-cell dataset GSE214695 (comprising 12 CD colon samples) [36] for analysis. We performed rigorous data curation and downstream analysis of 46,700 single cells using Seurat R package (v5.3.0). Samples were then pooled together in the same object. Low-quality cells were then filtered out based on mitochondrial RNA percentage and number of genes per cell. Immunoglobulin (IG) genes were removed from all the main cell types except B and plasma cells to reduce background noise. Principal component analysis (PCA) was conducted to assess the variation and distribution patterns of the dataset. Dimensionality reduction was conducted by using the Uniform Manifold Approximation and Projection (UMAP) algorithm using the optimal number of PCs [37]. Marker genes were utilized to define each subcluster within the main cell types. The FindAllMarkers function was used to identify marker genes using the default threshold parameters, excluding the min.pct and the thresh.use, both of which were set to 0.25.

### 2.11. Statistical Analysis

All analyses in this study were conducted using the R software (v4.4.2), with statistical significance defined as *p* < 0.05.

## 3. Results

### 3.1. Detection of Co-DEGs Between IBD and MDD

**Figure 1:**
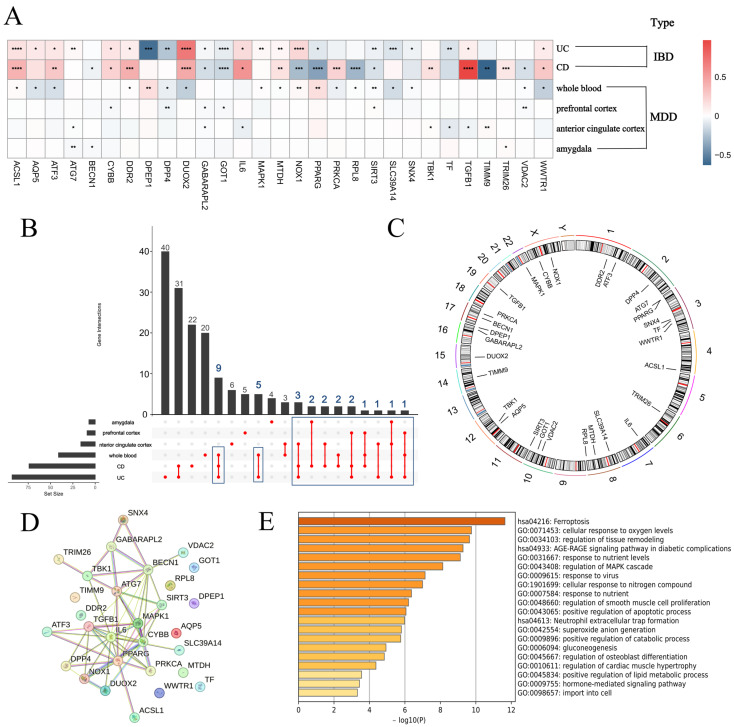
Identification of Co-DEGs associated with UC, CD, and MDD. (**A**) Heatmap of the expression profiles of 29 Co-DEGs in UC, CD, whole blood, prefrontal cortex, anterior cingulate cortex, and amygdala samples. (**B**) Upset plot illustrating the distribution of gene expression in multi-omics data. (**C**) Circos diagram showing the positions of Co-DEGs in the chromosome. (**D**) PPI network for 29 Co-DEGs. (**E**) GO annotation plot of the 29 Co-DEGs (*: *p* < 0.05, **: *p* < 0.01, ***: *p* < 0.001, ****: *p* < 0.0001).

This study investigated whether ferroptosis could act as a common regulatory mechanism in the pathogenesis of IBD and MDD by identifying Co-DEGs linked to both dis-eases. Gene expression data were obtained from the GEO database, including UC colon, CD colon, MDD whole blood, and MDD prefrontal cortex, anterior cingulate cortex, and amygdala samples. Differential expression analysis between normal and diseased samples revealed 29 Co-DEGs consistently dysregulated in both IBD and MDD. A heatmap was used to visualize the expression patterns of these Co-DEGs across various sample types (Figure 1A), while an upset plot was employed to present the expression frequencies of DE-FRGs (Figure 1B). Furthermore, a Circos plot was constructed to provide a comprehensive overview of the expression profiles of the identified Co-DEGs (Figure 1C). Meanwhile, protein–protein interaction (PPI) analysis via the STRING database revealed potential interaction relationships among the 29 Co-DEGs, which facilitated the construction of a PPI network (Figure 1D). Finally, GO annotation of the 29 Co-DEGs was performed using the Metascape database (Figure 1E).

### 3.2. Correlation Analysis of the 29 Co-DEGs

**Figure 2:**
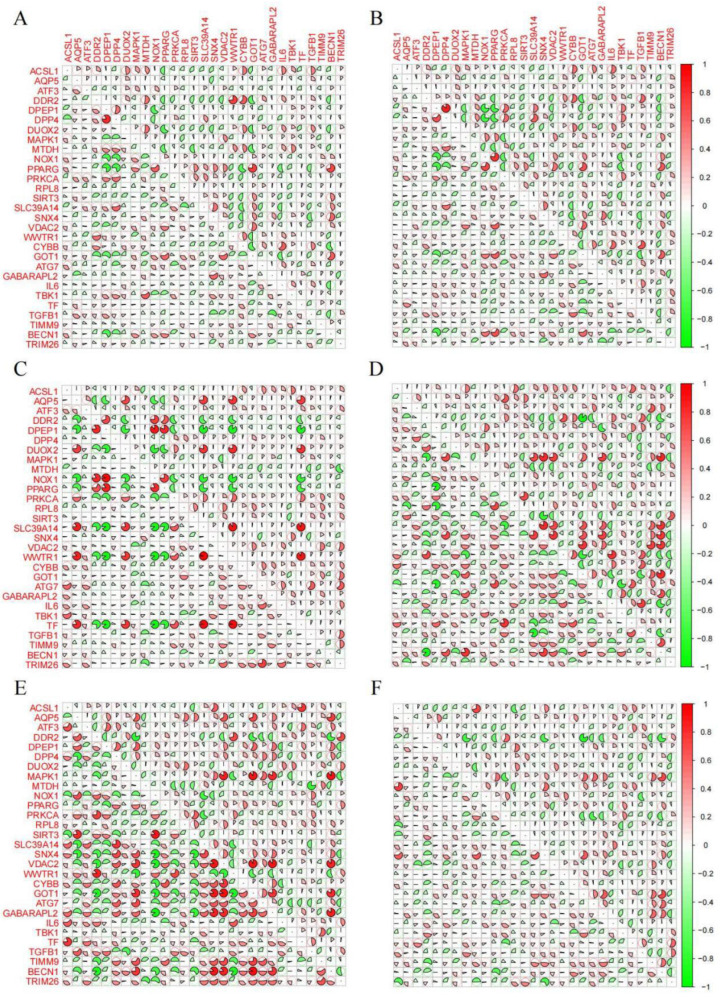
Correlation analysis of 29 Co-DEGs in control group and disease group. (**A**) UC: merged GSE13367, GSE24287, and GSE179285. (**B**) CD: merged GSE20881, GSE24287, and GSE179285. (**C**) MDD whole blood: merged GSE98793 and GSE19738. (**D**) MDD prefrontal cortex: GSE54571. (**E**) MDD anterior cingulate cortex: GSE54571. (**F**) MDD amygdala: GSE54564.

To precisely analyze the possible interacting network of Co-DEGs in both normal and disease groups, we performed comprehensive correlation analyses of these genes within each group. The correlation patterns of Co-DEGs in both disease and control groups were shown in Figure 2A–F. The lower-left section of the figures corresponds to the control group, whereas the upper-right section represents the disease group. The 29 Co-DEGs exhibit variable correlation profiles across different sample types. For instance, in the prefrontal cortex samples from patients with depression, the correlations among the 29 Co-DEGs were significantly stronger in the disease group than in the control group. In contrast, in the anterior cingulate cortex samples from depressed patients, the correlations among these Co-DEGs were significantly higher in the control group compared to the disease group. These analyses revealed significant correlations among Co-DEGs across various sample groups and provided insights into the potential interaction relationships of Co-DEGs.

### 3.3. Correlation Analysis Between 29 Co-DEGs and Immune Cells

**Figure 3:**
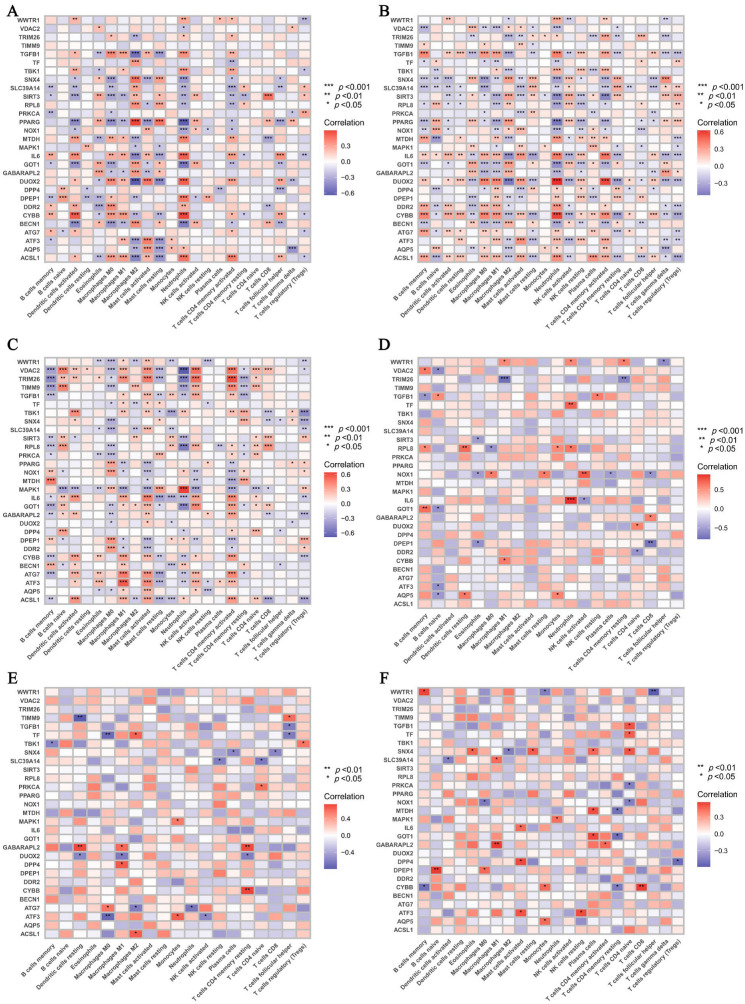
The correlations between 29 Co-DEGs and immune cell infiltration. The heatmap shows the correlation of Co-DEGs with immune cells in (**A**) UC colon samples, (**B**) CD colon samples, (**C**) MDD whole blood samples, (**D**) MDD prefrontal cortex samples, (**E**) MDD anterior cingulate cortex samples, and (**F**) MDD amygdala samples.

We analyzed the relationships between 29 Co-DEGs and immune cell infiltration in UC colon, CD colon, MDD whole blood, and MDD prefrontal cortex, anterior cingulate cortex, and amygdala samples (Figure 3A–F). Multiple immune cell types showed significant correlations with Co-DEGs in both IBD and MDD. For example, memory B cells were negatively correlated with SIRT3 expression in UC colon and MDD whole blood samples. These findings suggest that Co-DEGs may contribute to IBD and MDD pathogenesis by modulating the infiltration of specific immune cells through positive or negative regulatory effects.

### 3.4. Machine Learning Screening for the Important Genes

**Figure 4:**
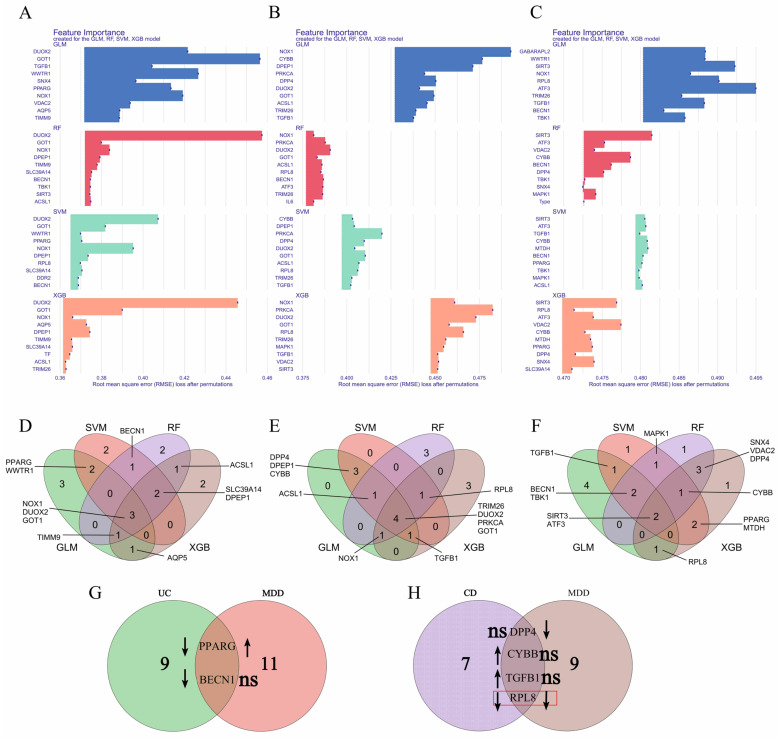
Important Co-DEGs associated with UC, CD, and MDD as identified by machine learning algorithms. The top 10 Co-DEGs associated with UC colon samples (**A**), CD colon samples (**B**), and MDD whole blood samples (**C**) identified by GLM, RF, SVM, and XGB. Wayne diagram of important genes shared by two or more machine learning algorithms in UC colon samples (**D**), CD colon samples (**E**), and MDD whole blood samples (**F**). (**G**) Venn diagram of the intersection between UC and MDD. (**H**) Venn diagram of the intersection between CD and MDD. • represents the importance score. The arrows represent the changing trends of genes in these diseases.

Owing to the limited sample sizes for MDD prefrontal cortex, anterior cingulate cortex, and amygdala, the machine learning models showed reduced accuracy. Therefore, we focused on colon tissue from UC and CD patients and whole blood from MDD patients for further analysis. Using SVM, RF, XGB, and GLM algorithms, we identified the top ten most significant Co-DEGs for UC (Figure 4A), CD (Figure 4B), and MDD whole blood (Figure 4C). ROC curves confirm that their machine learning-based gene prioritization is reliable (Supplementary Figure S2). A Venn diagram was used to identify genes shared by two or more machine learning algorithms for UC (Figure 4D), CD (Figure 4E), and MDD whole blood (Figure 4F). Subsequently, another Venn diagram was constructed to identify the common key genes shared between UC and MDD (Figure 4G) and between CD and MDD (Figure 4H). Among these overlapping genes, *RPL8* showed a consistent downregulation trend in both IBD and MDD disease samples; therefore, *RPL8* was selected as a potential biomarker.

### 3.5. Comparison and Analysis of RPL8+ and RPL8− Groups

**Figure 5:**
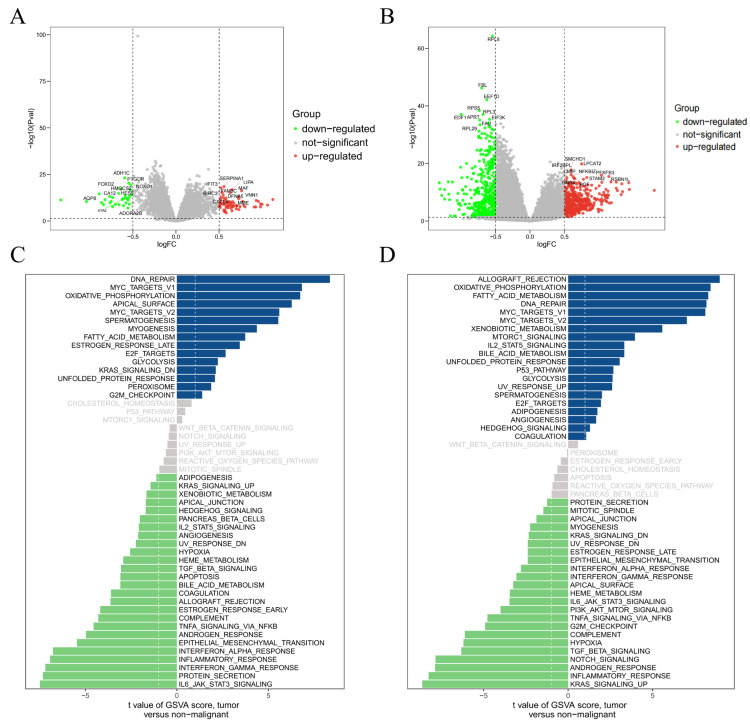
Identification of *RPL8+* and *RPL8−* groups. (**A**) The volcano plot DEGs identified between *RPL8+* and *RPL8−* groups (CD, *p* < 0.05). (**B**) The volcano plot DEGs identified between *RPL8+* and *RPL8−* groups (MDD whole blood, *p* < 0.05). (**C**,**D**) GSVA between *RPL8+* and *RPL8−* groups.

Since *RPL8* is an important disease-related gene with decreased expression in both CD samples and MDD whole blood samples, we sorted the samples by the expression level of *RPL8* from low to high. The first half of the samples were designated as the *RPL8−* group and the latter half as the *RPL8+* group. In the CD samples, a total of 99 DEGs were identified between the *RPL8+* and *RPL8−* groups, including 67 upregulated and 32 downregulated genes (Supplementary Table S2), while in the MDD whole blood samples, 949 DEGs were obtained between the two groups, comprising 422 upregulated genes and 527 downregulated genes (Supplementary Table S2) (Figure 5A,B). To better understand the functional implications of these DEGs, we performed GO and KEGG analyses for each of the four gene sets (Supplementary Figure S3). According to the results of GSVA, in CD samples, the *RPL8+* group was mainly enriched in pathways such as DNA repair, myc targets v1, and oxidative phosphorylation, whereas the *RPL8−* group was primarily enriched in IL6 jak stat3 signaling, protein secretion, and interferon gamma response (Figure 5C). In MDD whole blood samples, the *RPL8+* group was mainly enriched in allograft rejection, oxidative phosphorylation, and fatty acid metabolism, while the *RPL8−* group was mainly enriched in kras signaling, the inflammatory response, and the androgen response (Figure 5D).

### 3.6. Molecular Docking Analysis

**Figure 6:**
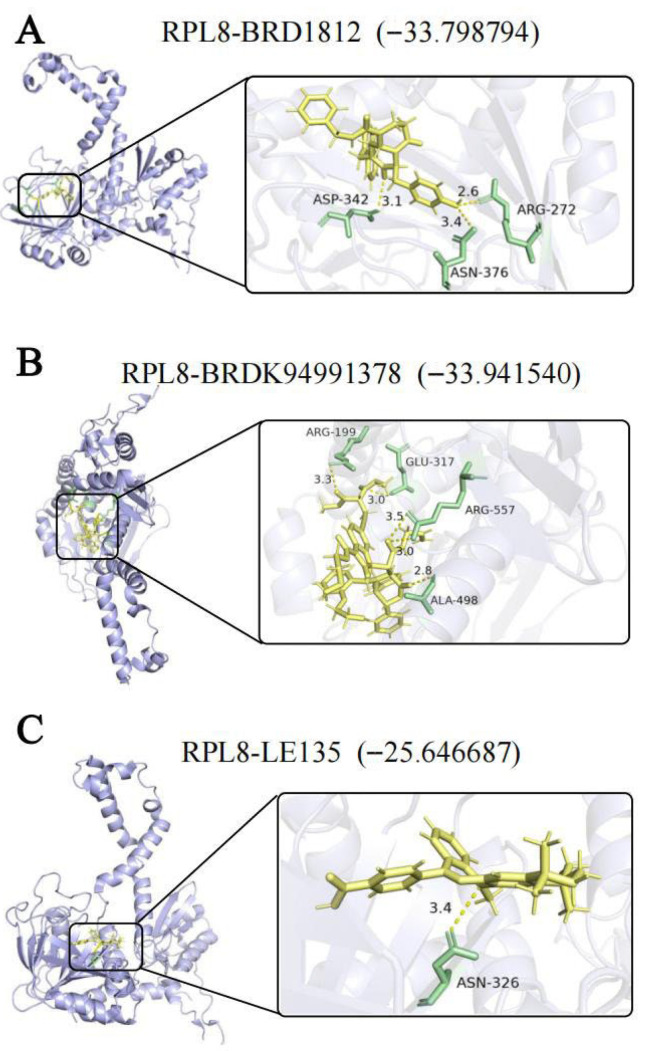
Molecular docking analysis. (**A**) *RPL8* bound with BRD1812. (**B**) *RPL8* bound with BRDK94991378. (**C**) *RPL8* bound with LE135.

Using the expression profiles of Co-DEGs linked to IBD and MDD, we predicted drugs for six sample types: UC colon, CD colon, MDD whole blood, and MDD prefrontal cortex, anterior cingulate cortex, and amygdala(Supplementary Figure S4). Among these predicted drugs, three were commonly identified across three or more sample types: BRD1812, BRDK94991378, and LE135 (Supplementary Table S3). Subsequently, molecular docking analyses were performed between the biomarker *RPL8* and these three candidate drugs. BRD1812 bound to *RPL8* through three hydrogen bonds with residues ASP-342, ASN-376, and ARG-272 (Figure 6A), while BRDK94991378 formed five hydrogen bonds with *RPL8*, involving ARG-199, GLU-317, ARG-557, and ALA-498 (Figure 6B). LE135 formed one hydrogen bond with *RPL8* at the ASN-326 residue (Figure 6C). The calculated binding free energy scores indicate that BRD1812 and BRDK94991378 exhibit strong binding affinities with *RPL8*.

### 3.7. Molecular Dynamics Simulation

**Figure 7:**
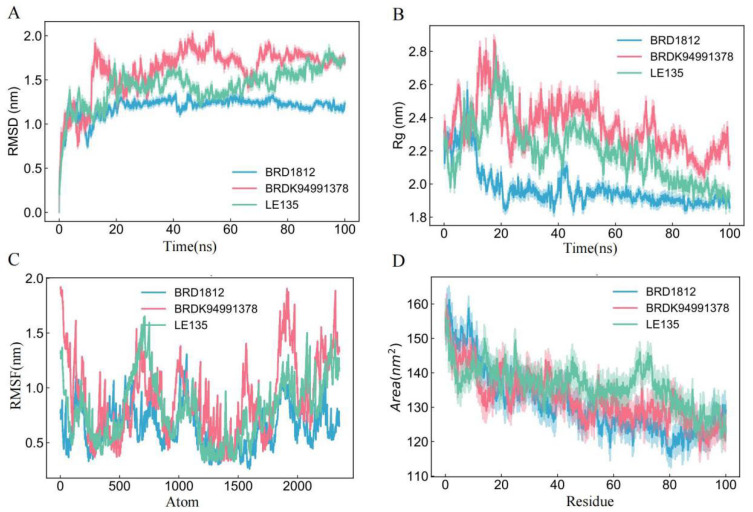
Molecular dynamics simulations of *RPL8* in complex with BRD1812, BRDK94991378, and LE135. Panels show (**A**) RMSD, (**B**) Rg, (**C**) RMSF, and (**D**) SASA analyses.

To further investigate the potential interacting networks between *RPL8* and potential therapeutic compounds, MDSs were conducted. During the simulations, the RMSD was calculated to assess structural changes relative to the reference structure over time (Figure 7A). Lower RMSD values for BRD1812 and LE135 indicate that these compounds maintained stable conformations closely aligned with the reference structure, whereas the higher RMSD observed for BRDK94991378 suggests greater structural deviation. The radius of gyration (Rg) was analyzed to evaluate the compactness of the molecules during the simulation (Figure 7B). BRD1812 and LE135 displayed consistently low Rg values, reflecting more compact structures, while BRDK94991378 exhibited higher Rg values, indicative of a more expanded conformation. Root-mean-square fluctuation (RMSF) analysis was performed to examine atomic-level flexibility (Figure 7C). Lower RMSF values suggest tighter packing within the protein–ligand complex, with *RPL8* showing particularly close interaction with BRD1812. Additionally, solvent-accessible surface area (SASA) was measured to determine the extent of molecular surface exposure to solvent (Figure 7D). All three compounds displayed similar SASA trends throughout the simulations. Collectively, these results indicate that BRD1812 demonstrates the most favorable binding stability and compact interaction with *RPL8*, highlighting its potential as a promising therapeutic candidate.

### 3.8. Spatial Mapping Profiling via Single-Cell Transcriptomic Datasets

**Figure 8:**
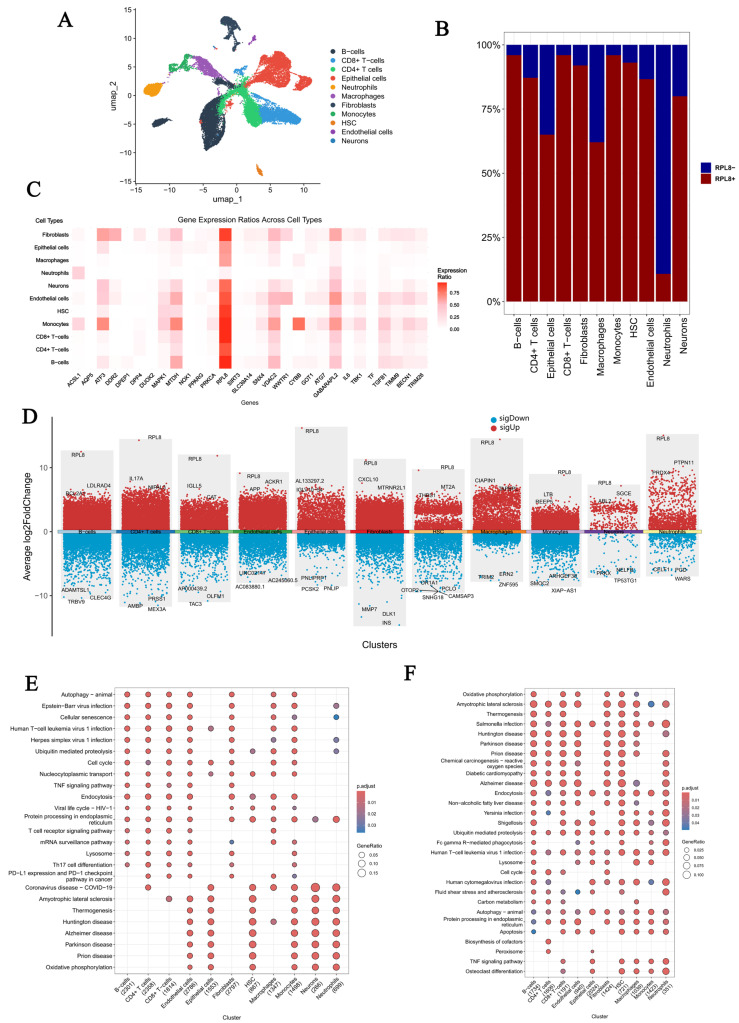
Single-cell sequencing analysis. (**A**) Cell type annotation. (**B**) Bar plot showing the distribution of *RPL8*+ and *RPL8*− cells across clusters. (**C**) Heatmap illustrating the distribution of 29 DE-FRGs across different cell types. (**D**) Scatter plot of differential expression analysis between *RPL8*+ and *RPL8*− cells. (**E**) KEGG pathway enrichment of upregulated DEGs. (**F**) KEGG pathway enrichment of downregulated DEGs.

We selected the GSE214695 transcriptomic dataset for single-cell sequencing analysis. First, we extracted the top 1500 genes with the highest cell-to-cell coefficient of variation and performed dimensionality reduction using principal component analysis (PCA) (Supplementary Figure S5). Subsequently, we applied the UMAP algorithm for cell clustering and annotated the identified cell types (Figure 8A). We examined the distribution of *RPL8+* and *RPL8−* cells across 11 distinct cell types (Figure 8B), revealing that *RPL8−* cells accounted for a large proportion of neutrophils. We then investigated the distribution of 29 Co-DEGs across these 11 cell types (Figure 8C), followed by a differential analysis between *RPL8+* and *RPL8−* cells (Figure 8D). The resulting upregulated and downregulated DEGs were subjected to KEGG pathway analysis. A comparison of the results revealed several overlapping pathways, including Amyotrophic lateral sclerosis, Thermogenesis, Huntington disease, Alzheimer disease, Parkinson disease, Prion disease, Oxidative phosphorylation, Human T-cell leukemia virus 1 infection, Lysosome, TNF signaling pathway, Endocytosis, Ubiquitin-mediated proteolysis, Cell cycle, Autophagy—animal, and Viral life cycle (Figure 8E,F).

**Figure 9:**
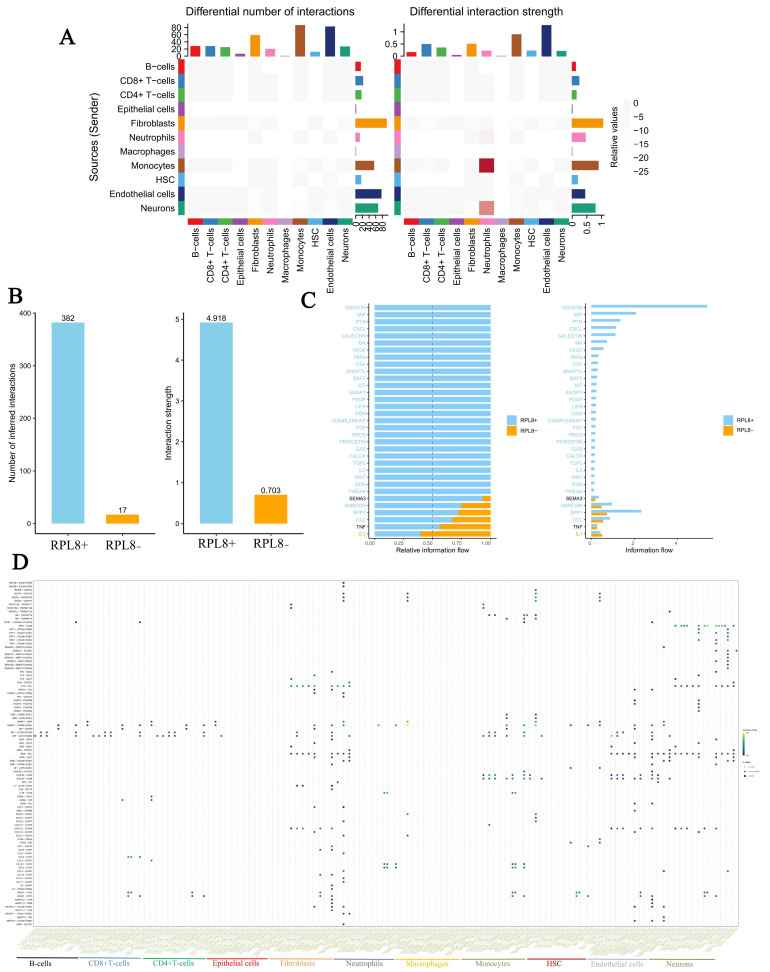
Cell–cell communication between *RPL8*+ and *RPL8*− cells. (**A**) Differential interactions between cell types. (**B**) Bar plot comparing the inferred number and strength of interactions between *RPL8*+ and *RPL8*− cells. (**C**) Visualization of differences in molecular information flow. (**D**) Bubble plot of cell–cell communication.

Subsequently, we explored the differences in cell–cell communication between *RPL8+* and *RPL8−* cells. Interaction analysis among different cell types revealed a marked increase in the number of interactions involving macrophages, suggesting a potentially more active involvement of macrophages in signal transduction, immune regulation, and processes related to inflammation and tissue repair during disease progression (Figure 9A). Further comparison of both the frequency and intensity of these interactions indicated that *RPL8+* cells exhibited markedly higher interaction activity, pointing to substantial differences in communication-related properties between the two groups (Figure 9B). Molecular information flow analysis (Figure 9C) showed that, in the left panel, key signaling molecules such as KIT, ANGPT, and PDGF were predominantly represented in blue (High), indicating that they serve as “core carriers” in intercellular communication and play a dominant role in signal transmission. In contrast, molecules such as SEMA3, ANNEXIN, SPP1, TNF, and IL1 had a higher proportion of orange (Low), suggesting that the communication mediated by these molecules is more prominent in the Low group. Finally, we compared the cell–cell communication patterns of *RPL8+* and *RPL8−* cells using a bubble plot representation of intercellular interactions (Figure 9D).

## 4. Discussion

IBD is a refractory inflammatory disease of the gastrointestinal tract with an unclear cause. Its pathogenesis is considered multifactorial, with current hypotheses proposing that interactions between the gut microbiome and environmental factors trigger ab-normal mucosal immune responses in genetically predisposed individuals [38]. Notably, gut microbiome dysbiosis has been implicated in the pathogenesis of IBD, contributing to exaggerated immune responses and impaired intestinal barrier integrity [39]. Growing evidence further suggests that the gut microbiota is closely linked to a wide range of diseases. In this context, microbiota-derived metabolites serve as key mediators, interacting with multiple host systems to regulate physiological and pathological processes [40]. Ferroptosis is a unique, iron-dependent form of non-apoptotic cell death, characterized by uncontrolled and overwhelming peroxidation of polyunsaturated fatty acids in membrane phospholipids, eventually causing plasma membrane rupture [41,42]. Ferroptosis displays a morphology distinct from other forms of cell death, including apoptosis, necroptosis, and pyroptosis. As an important cell death pathway, it affects diverse cell types—such as neurons, renal tubular epithelial cells, endothelial cells, and T cells—and contributes to the development of various diseases linked to cellular dysfunction [15,43]. In recent years, increasing evidence has shown that ferroptosis is involved in the pathophysiology of IBD. However, its precise roles and underlying molecular mechanisms have not been fully elucidated [44]. Previous experimental studies have demonstrated that seliciclib alleviates ulcerative colitis (UC) by inhibiting ferroptosis and reducing intestinal inflammation [45]. In contrast, isorhamnetin was reported to exacerbate colitis by inducing ferroptosis, possibly through activation of the NRF2/HO-1 pathway and iron chelation [46]. Additionally, butyrate has been reported to shield cells from oxidative stress and potentially inhibit ferroptosis, evidenced by reduced levels of the ferroptosis marker H_2_O_2_ [47]. Conversely, other studies revealed that acrylamide (ACR) induces oxidative stress and triggers ferroptosis in Sertoli cells [48]. Therefore, ferroptosis is a promising therapeutic target for IBD treatment. It should be noted that the present study cannot fully determine whether the observed alterations in FRG expression serve as causal drivers of disease pathogenesis or are merely secondary consequences of the disease process. While our integrative bioinformatics analyses identified robust associations across independent datasets, the issue of causality remains unresolved. Further mechanistic studies, including functional experiments in cellular and animal models, are warranted to clarify the extent to which FRGs actively contribute to disease initiation and progression versus reflecting downstream pathological changes.

Major depressive disorder (MDD) is one of the most common psychiatric disorders, affecting nearly 20% of the global population and contributing substantially to morbidity, mortality, and socioeconomic burden. Its pathogenesis is multifactorial, with chronicity, systemic illness, and neuroinflammation identified as key contributors [49]. In the central nervous system (CNS), microglia—the resident macrophages—are critical for maintaining neural homeostasis [50,51]. Dysregulation of microglial function, together with immuno-metabolic disturbances, cognitive deficits, and structural brain abnormalities, has been increasingly implicated in the development and progression of MDD. Recent research has highlighted a possible link between ferroptosis and the onset of depressive disorders. Clinical and experimental evidence suggests that ferroptosis may influence the development and function of the hippocampus through interactions with neuroinflammatory pathways [52]. A increasing number of studies have demonstrated that the disruption of ferroptosis-associated phenomena, such as iron homeostasis and lipid ROS accumulation, can observed in neurological diseases [53,54,55,56]. These evidences suggest that ferroptosis may be involved in the progression of neuroinflammation-associated disorders including MDD [57].

Recent findings as to the gut–brain axis (GBA) suggest that GBA represents a complicated interplay between the gut and the central nervous system [58]. Earlier observational studies have indicated a two-way relationship between IBD and MDD [59]. Existing studies have indicated that FRGs are closely associated with the progression of both IBD and MDD. However, the expression patterns and potential regulatory mechanisms of FRGs have not yet been systematically compared between these two diseases. This study aimed to compare the expression patterns and underlying regulatory mechanisms of FRGs between IBD and MDD and to explore their potential role in the comorbidity of the two diseases. We identified 29 Co-DEGs in IBD and MDD: *ACSL1*, *AQP5*, *ATF3*, *DDR2*, *DPEP1*, *DPP4*, *DUOX2*, *MAPK1*, *MTDH*, *NOX1*, *PPARG*, *PRKCA*, *RPL8*, *SIRT3*, *SLC39A14*, *SNX4*, *VDAC2*, *WWTR1*, *CYBB*, *GOT1*, *ATG7*, *GABARAPL2*, *IL6*, *TBK1*, *TF*, *TGFB1*, *TIMM9*, *BECN1*, and *TRIM26*. Subsequently, we predicted drugs that might affect the expression of Co-DEGs, leading to the identification of three compounds that were common across three or more sample types: BRD1812, BRDK94991378, and LE135. Molecular docking and dynamics simulations with *RPL8*, a machine learning-identified biomarker, highlighted BRD1812 as the most promising candidate.

In this investigation, we initially examined the expression profiles of ferroptosis-related genes (FRGs) and detected 29 co-differentially expressed genes (Co-DEGs) that exhibited consistent dysregulation in both inflammatory bowel disease (IBD) and major depressive disorder (MDD). PPI analysis revealed significant interconnectivity among key regulatory genes, including *ACSL1*, *ATF3*, *DPP4*, *DUOX2*, *MAPK1*, *NOX1*, *PPARG*, *PRKCA*, *SIRT3*, *SLC39A14*, *SNX4*, *VDAC2*, *CYBB*, *ATG7*, *GABARAPL2*, *IL6*, *TBK1*, *TGFB1*, *BECN1*, and *TRIM26*, forming a cohesive functional module. Correlation analyses highlighted distinct co-expression patterns of Co-DEGs between control and disease groups, with particularly pronounced differences observed in prefrontal cortex and anterior cingulate cortex samples from MDD patients. Given that numerous immune-related cells, such as regulatory T cells, play a crucial role in the regulation of anti-inflammatory and neurotrophic functions [60], we then explore immune regulation by analyzing the relationship between Co-DEGs/DE-FRGs and immune cell infiltration. Our earlier analyses demonstrated notable alterations in immune cell composition. Specifically, naïve B cells, γδ T cells, and M2 macrophages were markedly diminished in IBD and MDD cohorts relative to healthy controls, while resting NK cells, monocytes, and M1 macrophages showed significant enrichment. Additionally, plasma cells exhibited an opposite trend, being elevated in CD and MDD whole-blood samples but reduced in the MDD amygdala [61]. The findings indicated that these genes are strongly linked to immune activity at inflammatory sites, influencing the composition and distribution of various immune cell subsets. Subsequently, machine learning methods were employed to identify *RPL8* as a potential diagnostic biomarker. Molecular docking and molecular dynamics simulations between *RPL8* and candidate drugs targeting Co-DEGs identified BRED1812 as the most promising therapeutic agent with dual efficacy for both IBD and MDD. Finally, single-cell RNA sequencing was performed to cluster and annotate cell populations within CD samples, followed by comparative analysis of gene expression differences between *RPL8+* and *RPL8−* cell subsets.

*RPL8*, a ribosomal protein involved in protein synthesis and cellular homeostasis, has recently attracted attention for its potential roles beyond ribosome function. Previous studies have shown that silencing *RPL8* inhibits the progression of hepatocellular carcinoma by downregulating the mTORC1 signaling pathway [62]. Other research has also suggested that *RPL8* may be a potential key regulatory factor in the pathogenesis of cleft palate [63]. Emerging evidence suggests that dysregulation of ribosomal proteins may contribute to inflammatory responses, cellular stress, and neurobiological processes. In this context, our findings highlight *RPL8* as a candidate biomarker that may link inflammatory bowel disease and depression. Although direct mechanistic studies are still lacking, the identification of *RPL8* in our analysis suggests that alterations in fundamental cellular processes, such as ribosomal function and protein translation, could represent a convergent pathway underlying both intestinal and neuropsychiatric pathology. This underscores the potential importance of *RPL8* as not only a diagnostic marker but also a promising therapeutic target warranting further investigation. Although our analysis identified *RPL8* as a potential key biomarker linking inflammatory bowel disease and depression, it should be acknowledged that the current biological evidence supporting this association remains limited. The role of *RPL8* in neuropsychiatric or inflammatory pathways has not been well established, and our findings are primarily based on computational predictions. Therefore, while the results are intriguing, they should be interpreted with caution. Further experimental studies, particularly mechanistic investigations in cellular and animal models, are required to validate the biological relevance of *RPL8* and to elucidate its potential contribution to disease pathogenesis. Functional analyses from previous studies have shown that *RPL8* is involved in angiogenesis and immune system pathways [64]; however, to the best of our knowledge, no previous studies have directly linked *RPL8* to neural pathways or neuropsychiatric disorders. Due to the lack of studies linking *RPL8* to immune or neural pathways, we conducted correlation analyses between *RPL8* and immune as well as neural pathways in different samples (Supplementary Figure S6). The results showed that in IBD samples, *RPL8* was positively correlated with both immune and neural pathways, while in MDD samples, *RPL8* was negatively correlated with both immune and neural pathways. Therefore, our findings provide novel evidence that *RPL8* may serve as a shared biomarker and a promising therapeutic target bridging inflammatory bowel disease and depression, although further mechanistic investigations are clearly warranted.

These advances enhance our cross-disciplinary understanding of the shared mechanisms between IBD and MDD and offer valuable experimental evidence and translational perspectives for therapies addressing both gut inflammation and emotional dysregulation. Moreover, they support the identification of cross-disease biomarkers with both diagnostic and prognostic value. Collectively, these findings may help shift the clinical management of IBD and MDD beyond their traditionally siloed approaches, toward a more integrated and personalized model of coordinated intervention.

A number of limitations should be acknowledged in the present study. First of all, the sample size for MDD was relatively small. The relatively small sample size for MDD remains a key limitation, which may restrict the generalizability and robustness of the findings we observed. Further investigation with larger number of samples will be essential to further confirm these results. Although the available data partially reflect key features of the disorder, the limited sample size may constrain the generalizability and robustness of the findings. Future studies should incorporate larger and more diverse cohorts to enhance the reliability and validity of the conclusions. Second, while we identified 29 Co-DEGs that are commonly dysregulated across multiple conditions, these findings are based on preliminary computational predictions and currently lack experimental validation in vitro or in vivo. The biological relevance and functional roles of these genes must be further confirmed through rigorous experimental approaches. We plan to generate *RPL8* knockout and wild-type mouse models and to evaluate a range of molecular, histological, and behavioral indicators to investigate their roles in the pathogenesis of IBD and MDD. We also plan to establish mouse models of colitis, depression, and comorbid disease in future studies. Using these models, we will investigate the expression changes in *RPL8* in different tissues across disease and control groups through qPCR and Western blot analyses. Additionally, although BRD1812 was identified Via bioinformatic analyses as a potential therapeutic candidate for both IBD and MDD, its molecular effects and underlying mechanisms of action at the cellular and organismal levels remain unexplored. Since BRD1812 is still at an early data stage and publicly available information is very limited, we can only rely on molecular docking and MD simulations to predict its potential therapeutic effects on IBD and MDD. Future work will involve comprehensive cellular functional assays to experimentally confirm the therapeutic effects and molecular mechanisms of BRD1812. Therefore, a series of comprehensive functional experiments—including both in vitro and in vivo studies—are necessary to elucidate the mechanistic link between the drug’s action and its biological phenotypes. These efforts will strengthen the credibility of our observations and offer critical insights for future research in this field.

## 5. Conclusions

In this study, 29 Co-DEGs were identified as being differentially expressed in both IBD and MDD. These results suggest possible pathological connections between the two disorders, offering a foundation for interdisciplinary research and advancing insight into mind–body interactions. Through machine learning, six disease-related characteristic genes were identified. Among these, *RPL8* was selected as a potential biomarker due to its consistent trend of changes in both CD and MDD. With *RPL8* as the target, BRD1812 was identified as the most promising drug. Importantly, monitoring specific gene expression levels may enable the early identification of IBD and MDD prior to the appearance of clinical symptoms, supporting timely diagnosis and intervention.

## Data Availability

mRNA expression profiles were obtained from the GEO database (https://www.ncbi.nlm.nih.gov/geo/ accessed on 1 July 2025).

## Supplementary Materials

The following supporting information can be downloaded at: https://www.mdpi.com/article/10.3390/genes16091111/s1, Figure S1: PCA plots of data before and after batch correction; Figure S2: ROC curves indicate the accuracy of gene prioritization via machine learning; Figure S3: GO analysis and KEGG analysis; Figure S4: Drug sensitivity thermogram; Figure S5:PCA plot of single-cell analysis; Figure S6: The correlation between *RPL8* and the neural pathway. Table S1: Ferroptosis-promoting genes list; Table S2: Differentially expressed gene between *RPL8−* group and *RPL8+* group; Table S3: The situation of shared drugs among different samples.
