# Supplementary material for: Machine Learning Identifies Shared Regulatory Mechanisms of Genes Associated with Ferroptosis in Major Depressive Disorder and Inflammatory Bowel Disease
Source: Genes (Basel). 2025 Sep 19;16(9):1111. doi: 10.3390/genes16091111 (PMC12470012; doi:10.3390/genes16091111)
Supplement: Supplementary file 1 [file genes-16-01111-s001.zip › Supplementary Figures.pdf]

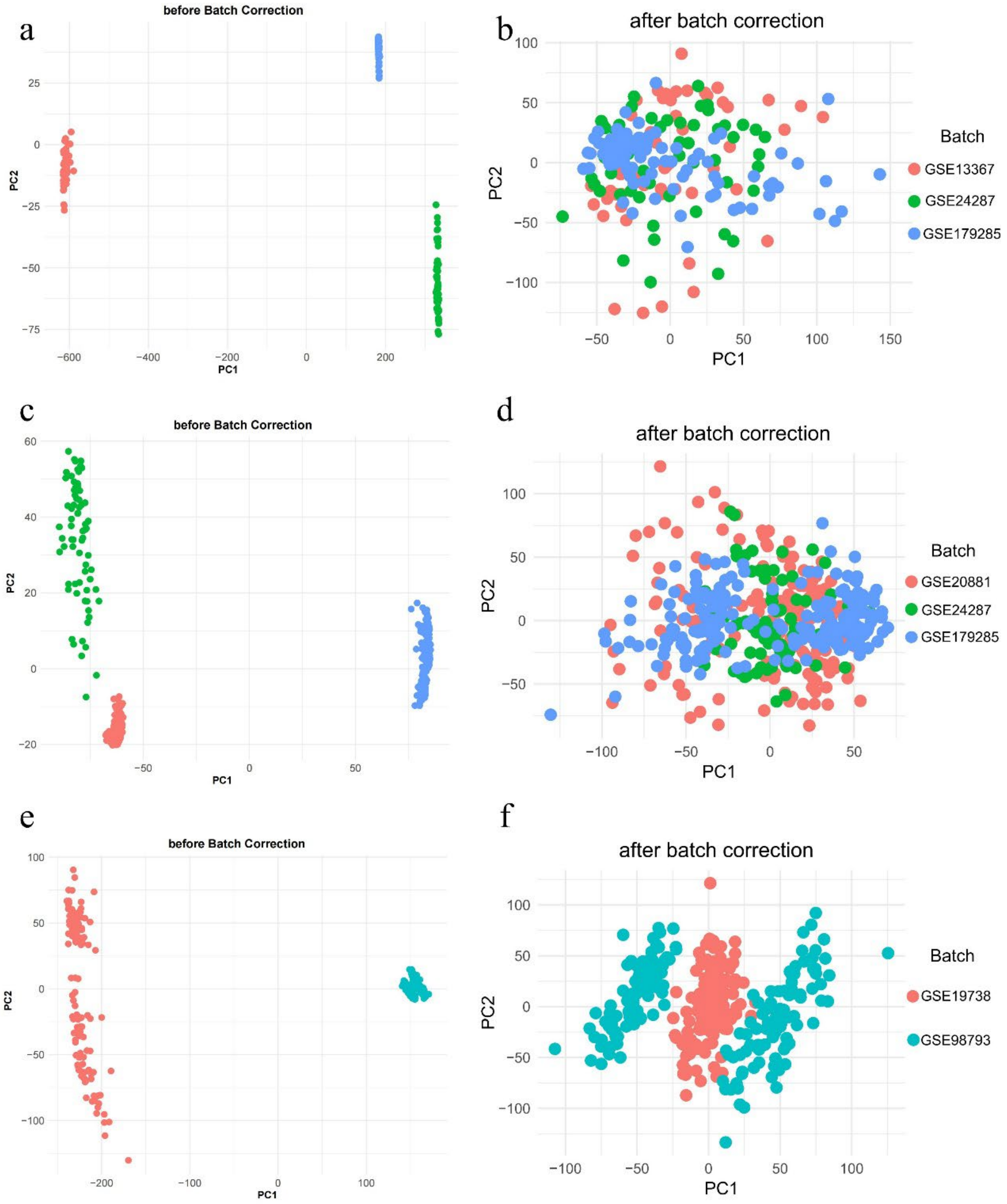

Supplementary Figure S1. PCA plots show the fixation before batch processing in the UC (a), CD (c), and MDD (e) datasets, and the fixation after batch processing in the UC (b), CD (d), and MDD (f) datasets.

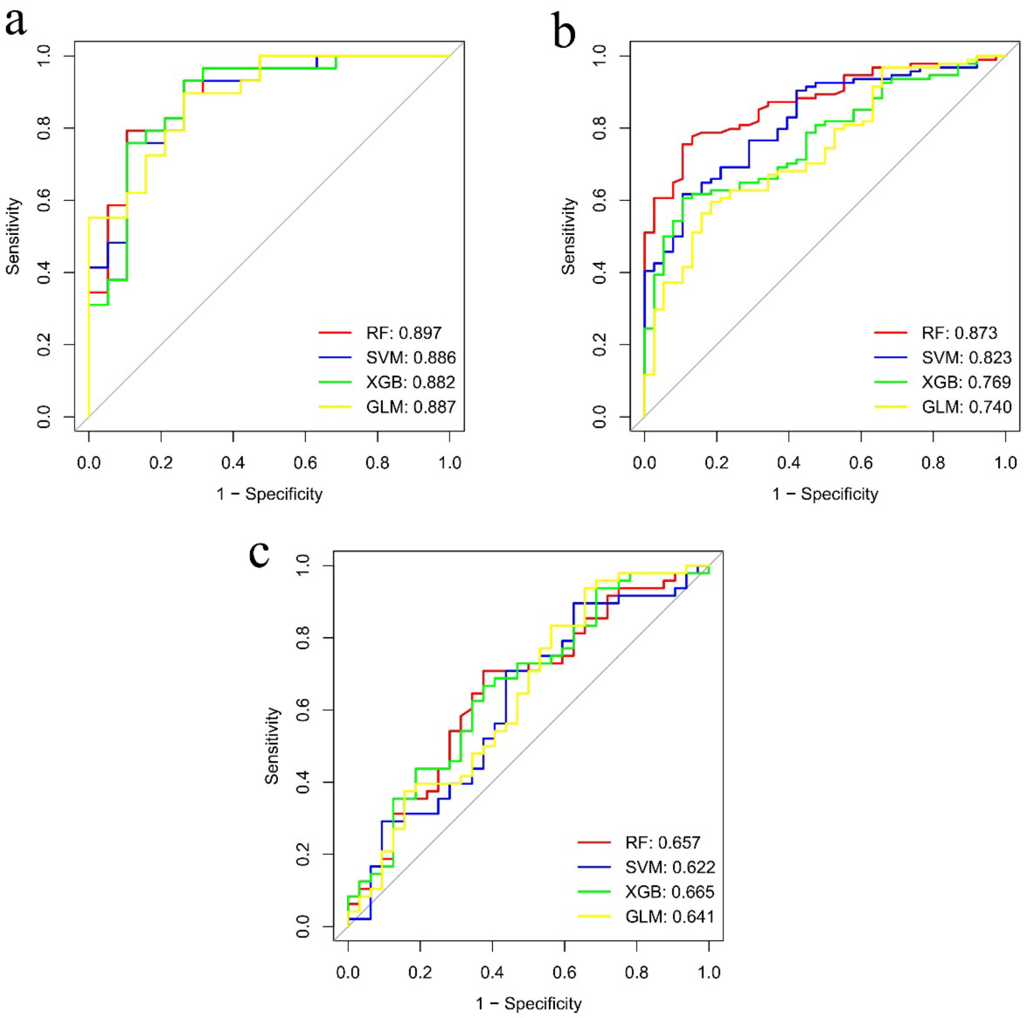

Supplementary Figure S2. In UC colon samples(**a**), CD colon samples(**b**), MDD whole blood samples(**c**), ROC curves indicate the accuracy of gene prioritization via machine learning.

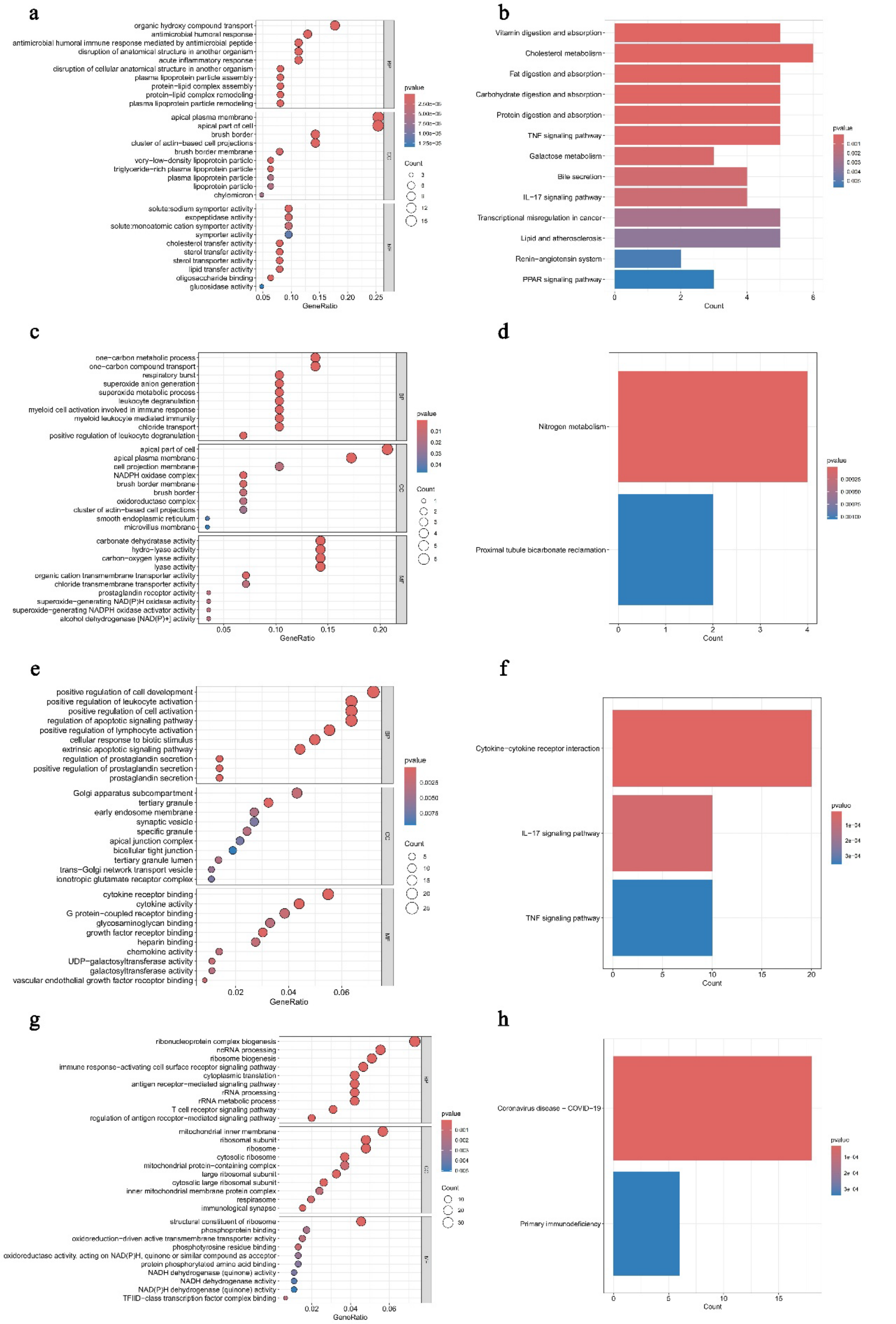

Supplementary Figure S3. In CD colon samples, GO analysis (**a**) and KEGG analysis (**b**) of 67 upregulated genes, and GO analysis (**c**) and KEGG analysis (**d**) of 32 downregulated genes. In MDD whole blood samples, GO analysis (**e**) and KEGG

analysis (**f**) of 422 upregulated genes, and GO analysis (**g**) and KEGG analysis (**h**) of 527 downregulated genes.

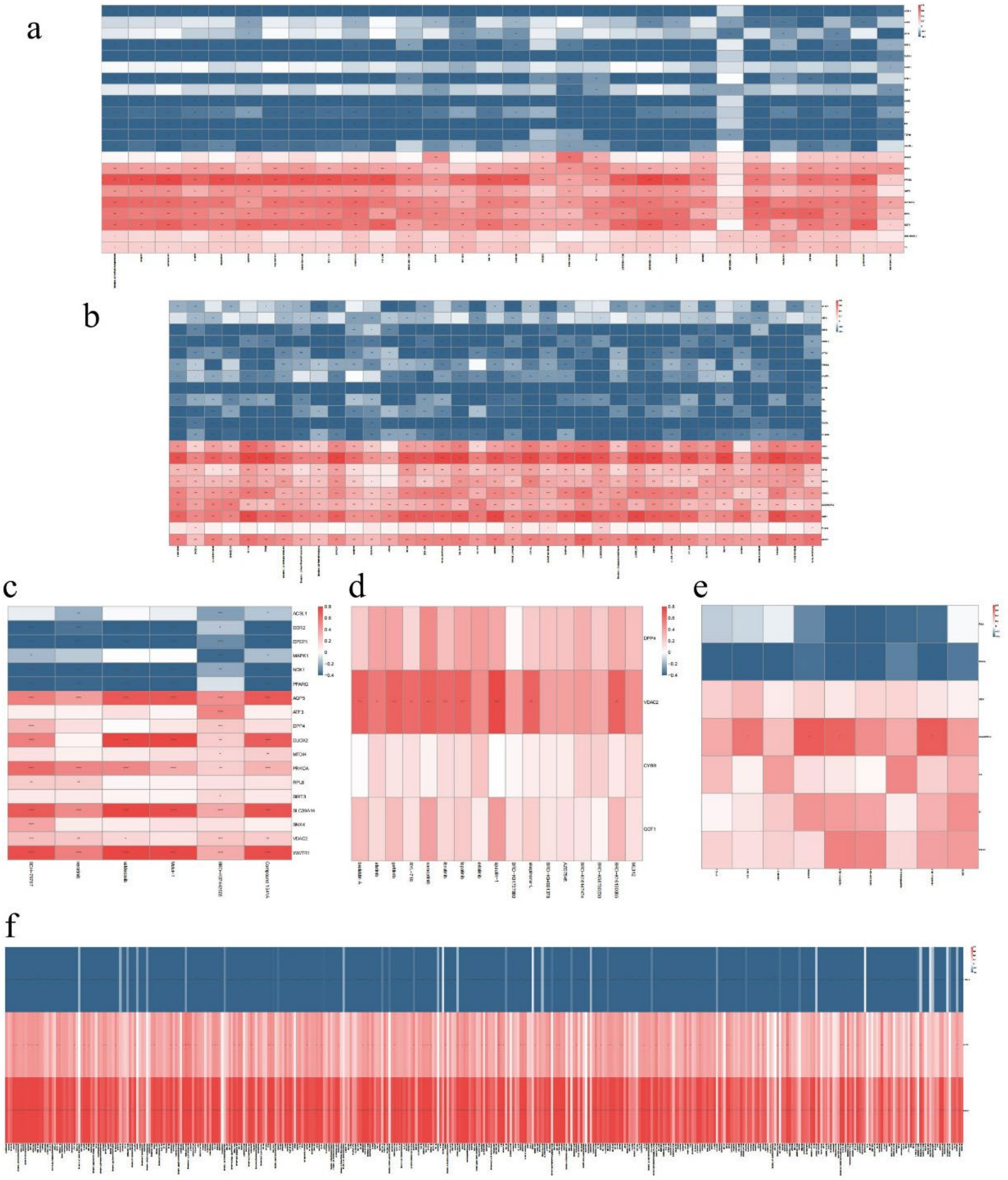

Supplementary Figure S4. a-f Drug sensitivity thermogram of UC colon samples, CD colon samples, MDD whole blood samples, MDD prefrontal cortex samples, MDD precingulate cortex samples and MDD amygdala samples.

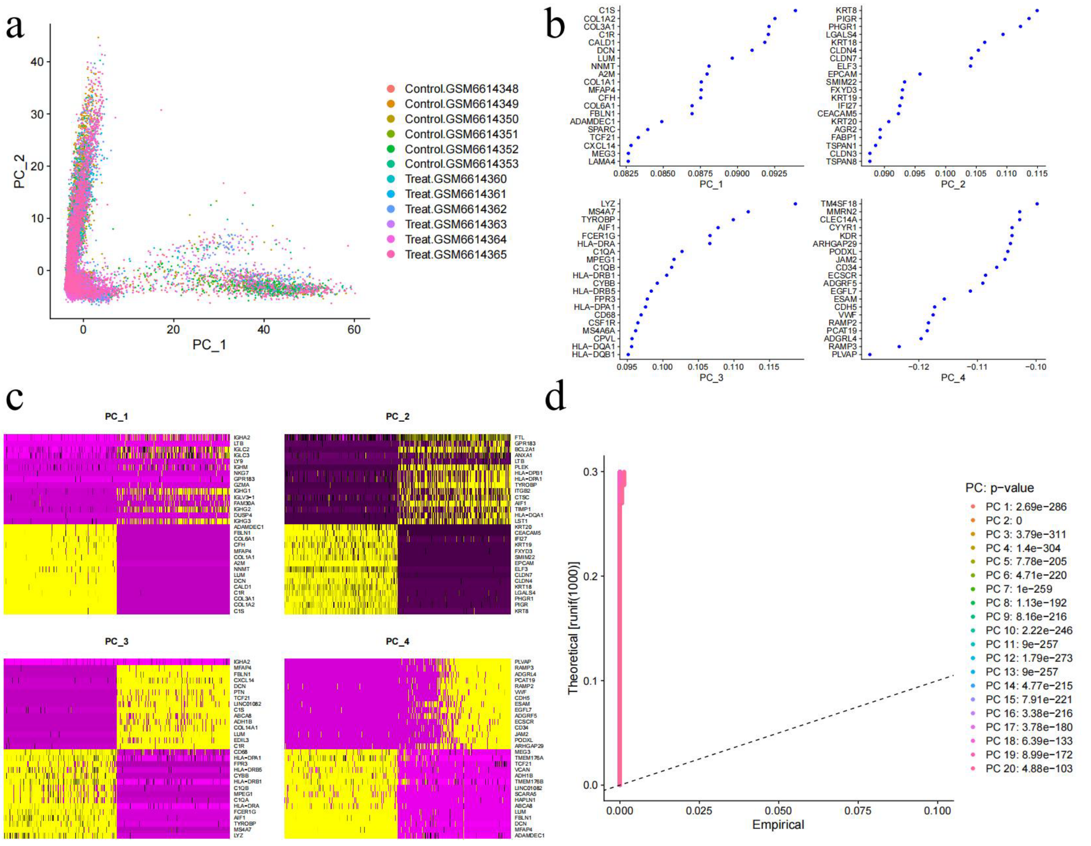

Supplementary Figure S5. (**a**). PCA scatter plot (**b**). PCA component characteristic gene distribution plot (**c**). PCA heatmap d. P-value distribution plot of PC

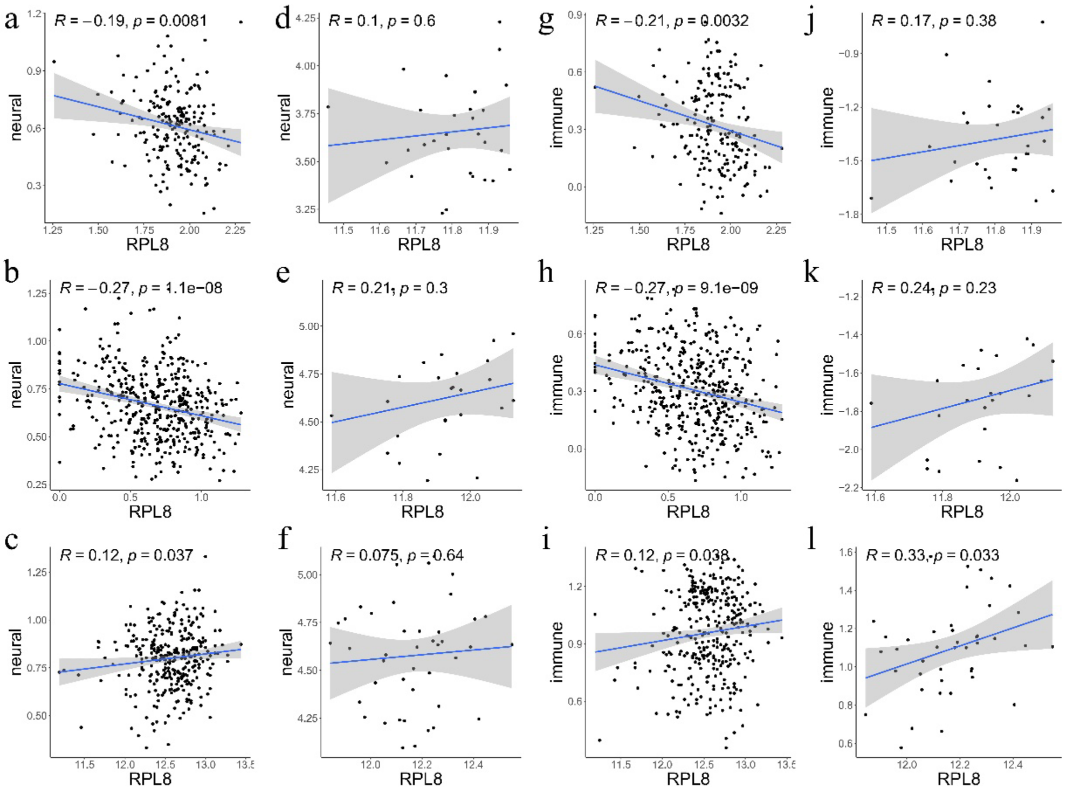

Supplementary Figure S6. The correlation between RPL8 and the neural pathway as well as the immune pathway in UC colon samples (a) (g), CD colon samples (b) (h), MDD whole blood samples (c) (i), MDD prefrontal cortex samples (d) (j), MDD precuneus cortex samples (e) (k), and MDD amygdala samples (f) (l).
